# Cell-to-Cell Natural Transformation Mediated Efficient Plasmid Transfer Between *Bacillus* Species

**DOI:** 10.3390/ijms26020621

**Published:** 2025-01-13

**Authors:** Chao Wang, Rui Zhao, Wenjie Yang, Wanting Jiang, Hao Tang, Shishen Du, Xiangdong Chen

**Affiliations:** State Key Laboratory of Virology, College of Life Sciences, Wuhan University, Wuhan 430072, China; wangchaobio@whu.edu.cn (C.W.);

**Keywords:** HGT, *Bacillus subtilis*, transformation, plasmid, wild *bacilli*

## Abstract

Horizontal gene transfer (HGT) plays a pivotal role in bacterial evolution, shaping the genetic diversity of bacterial populations. It can occur through mechanisms such as conjugation, transduction, and natural transformation. *Bacillus subtilis*, a model Gram-positive bacterium, serves not only as a robust system for studying HGT but also as a versatile organism with established industrial applications, such as producing industrial enzymes, antibiotics, and essential metabolites. In this study, we characterize a novel method of plasmid transfer, termed Cell-to-Cell Natural Transformation for Plasmid Transfer (CTCNT-P), which efficiently facilitates plasmid transfer between naturally competent *B. subtilis* strains. This method involves co-culturing donor and recipient cells under antibiotic stress and achieves significantly higher efficiency compared to traditional methods such as Spizizen medium or electroporation-mediated transformation. Importantly, we demonstrate that CTCNT-P is applicable for plasmid transformation in wild *B. subtilis* isolates from natural environments and other *Bacillus* species, including *Bacillus amyloliquefaciens*, *Bacillus licheniformis*, and *Bacillus thuringiensis*. The simplicity and efficiency of CTCNT-P highlight its strong potential for industrial applications, including genetic modification of wild *Bacillus* strains for synthetic biology and the development of biocontrol agents.

## 1. Introduction

Horizontal gene transfer (HGT) is a fundamental driver of bacterial and archaeal evolution, enabling the lateral transfer of genetic material between organisms regardless of their phylogenetic relationships [1,2]. In contrast to vertical inheritance, where genetic information is passed from parent to offspring, HGT allows genes to traverse species boundaries, contributing significantly to microbial evolution and adaptation. This phenomenon has garnered substantial interest in molecular biology, genomics, and microbiology due to its role in shaping the genetic diversity of life forms [3,4].

There are three classical pathways of horizontal gene transfer: conjugation [5], transduction [6], and natural transformation [7]. Conjugation involves the formation of physical connections, known as conjugative pili or sex pili, between donor and recipient bacterial cells. These structures enable the transfer of plasmids—circular DNA molecules that often carry diverse genetic cargos—from donor to recipient cells. Transduction relies on bacteriophages, viruses that infect bacteria, to mediate genetic exchange. During infection, bacteriophages may inadvertently package fragments of bacterial DNA, transferring these into recipient cells during subsequent infection cycles. This serendipitous process allows recipient bacteria to acquire traits such as antibiotic resistance or enhanced metabolic capabilities.

Natural transformation, on the other hand, involves the uptake of foreign DNA from the environment by competent bacterial cells, which can integrate the DNA into their genome or maintain it as extrachromosomal elements. Unlike conjugation and transduction, which are mediated by external agents (pili and bacteriophages, respectively), natural transformation is entirely controlled by the recipient cell, which encodes the machinery required for DNA uptake and internalization.

In addition to these well-characterized pathways, several noncanonical mechanisms of HGT have emerged over the past two decades, including gene transfer agents (GTAs), nanotubes, and membrane vesicles (MVs) [8,9,10]. Regardless of the mechanism, HGT plays a crucial role in microbial adaptation, enabling the acquisition of genes for antibiotic resistance, virulence, and novel metabolic functions, thereby enhancing genetic diversity and evolutionary fitness [1,3,11,12].

Natural transformation requires the recipient cell to develop natural competence, which is the ability to take up and process exogenous DNA. Approximately 80 bacterial species have been reported to exhibit natural competence [13]. The establishment of this competent state also referred to as the K-state, is influenced by various factors, including cell density, growth conditions, starvation stress, and exposure to antibiotics [13,14].

The DNA uptake process during natural transformation is relatively well characterized [13,15,16,17]. A DNA uptake pilus, which contains the DNA-binding protein ComEA, is essential for capturing DNA and transporting it to the periplasmic space, where it is further processed and traverses the cell membrane [17,18]. While there are slight differences between Gram-positive and Gram-negative bacteria in the details of this process, the overall mechanism appears to be conserved: double-stranded DNA (dsDNA) binds to the cell surface, is processed into single-stranded DNA (ssDNA), and is then delivered into the cytoplasm [10,17].

Many species within the genus *Bacillus*, particularly *Bacillus subtilis*, are widely used in both fundamental research and commercial applications. These applications encompass diverse fields such as cell morphogenesis, sporulation, motility, antibiotic resistance, biofilm formation, and genetic competence [18,19,20,21,22].

*Bacillus* strains are particularly valued for their exceptional ability to secrete large quantities of proteins (20–25 g/L) into culture media, making them a preferred choice for industrial production of degradative enzymes and other proteins [23]. Furthermore, their adaptability to diverse environmental conditions enables various *Bacillus* species to be used in agriculture as biological pesticides, where they control plant diseases, manage insect pests, and promote plant growth [24].

In both research and practical applications, genetic modification of *Bacillus* strains is often necessary to optimize their performance for specific purposes.

Several methods have been developed to introduce exogenous DNA into *Bacillus* strains, including conjugation [25], phage transduction [26], natural transformation [27], electroporation, and protoplast transformation. However, these approaches often face limitations when applied to environmental isolates, particularly certain wild-type *Bacillus subtilis* strains, where DNA introduction remains challenging.

We discovered that co-culturing two naturally competent *B. subtilis* strains with different genetic markers on selective Spizizen minimal medium (MM) plates resulted in the appearance of transformants at remarkably high frequencies, a phenomenon we termed cell-to-cell natural transformation (CTCNT) [28]. We demonstrated that CTCNT is significantly more efficient than traditional natural transformation assays using purified chromosomal DNA, which we referred to as DNA-to-cell natural transformation (DTCNT).

Moreover, we showed that CTCNT can facilitate the transfer of plasmid DNA, a process we call CTCNT-P [29]. Recent findings suggest that co-culturing donor and recipient *B. subtilis* cells enhances plasmid transfer efficiency, potentially through membranous structures that mediate genetic material exchange [30]. However, the CTCNT-P process remains poorly characterized, and it is unclear whether this method can be extended to other *Bacillus* species.

In this study, the focus was placed on characterizing the CTCNT-P process in *Bacillus subtilis* and other *Bacillus* species. The aim was to elucidate the factors influencing its efficiency, including donor-to-recipient cell ratios, co-culture duration, and stress conditions. Methodological approaches involved co-culture experiments to optimize transformation frequency, followed by comparative analyses with established methods such as electroporation [25] and Spizizen transformation [26]. Furthermore, the applicability of CTCNT-P was assessed in environmental isolates of *Bacillus*, exploring its potential as a simple and efficient tool for genetic modification of wild strains.

## 2. Results

### 2.1. Cell-to-Cell Natural Transformation Efficiently Promotes Plasmid Transfer in B. subtilis Strains

Cell-to-cell natural transformation (CTCNT) can mediate the transfer of both chromosomal DNA and plasmids, establishing it as a bidirectional gene transfer process [28]. To confirm that transformants obtained on selective plates arose specifically from plasmid transfer rather than chromosomal DNA transfer, we engineered a sextuple auxotrophic strain, designated TLPHMC-G, to serve as a universal donor for CTCNT-mediated plasmid transfer (CTCNT-P).

TLPHMC-G is derived from *Bacillus subtilis* 168 and carries deletions in six genes (*lysA*, *hisD*, *pheA*, *metC*, and *cysE*), which render it auxotrophic for essential amino acids. Additionally, the amyE locus was replaced with a cassette containing *Ppen-lacIΔ11-gfp-mut2*, preventing the production of amylase and thereby eliminating the strain’s ability to hydrolyze starch. This modification allows the origin of transformants to be verified based on the appearance of hydrolysis zones on starch-containing media.

We introduced plasmid pBE2 (6.3 kb), a non-conjugative shuttle vector conferring kanamycin resistance, into TLPHMC-G. For the CTCNT-P assay, TLPHMC-G/pBE2 served as the donor strain, and *B. subtilis* 168 as the recipient strain. Due to the multiple auxotrophic markers, the probability of TLPHMC-G cells spontaneously acquiring DNA to correct these deficiencies is negligible, ensuring its role solely as a donor strain.

**Figure 1:**
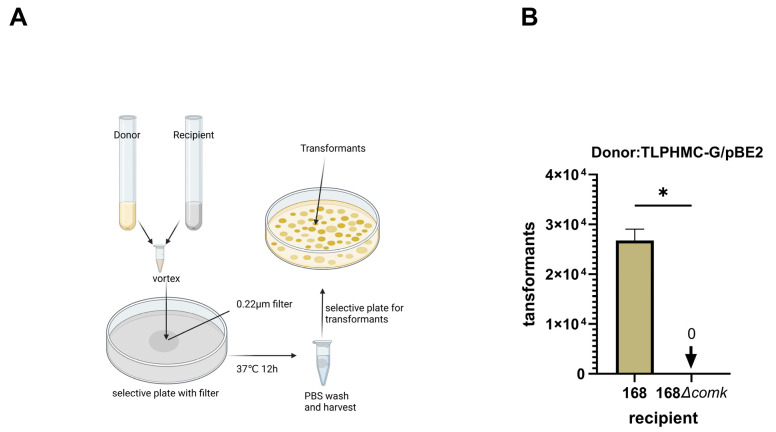
Cell-to-cell natural plasmid transformation between *B. subtilis*. (**A**) A workflow chart for plasmid transfer via CTCNT-P. (**B**) CTCNT-P is a natural transformation process: Deletion of the *comK* gene, which is essential for natural competence, resulted in no transformants, confirming that plasmid transfer is mediated by natural transformation. A t-test comparing Group 168 and Group 168Δ*comK* showed a significant difference (t(4) = 20.25, *p* = 0.0035). Data are presented as mean values with error bars representing the standard deviation (SD) (*n* = 3). Statistical significance is denoted as * *p <* 0.005.

The CTCNT-P protocol is summarized in Figure 1A: Briefly, the donor strain TLPHMC-G/pBE2 was diluted 1:100 (vol/vol) into fresh LB medium supplemented with kanamycin and incubated at 37 °C with shaking until the cell density reached approximately 1 × 10⁸ cells/mL. Concurrently, an overnight culture of the recipient strain was diluted into fresh minimal medium (MM) supplemented with tryptophan and incubated under identical conditions.

Equal volumes of donor and recipient cultures were then mixed and spotted onto a filter membrane (0.22 μm GSWP Mixed Cellulose Ester Membrane from Millipore) placed on a selective plate. This co-cultivation step facilitates the proliferation of transformants under selective conditions, increasing the likelihood of isolating successful transformants. After a 24 h incubation, the filter membrane was carefully removed and washed with PBS, and surviving cells were harvested, diluted, and plated onto a selective medium. Transformation efficiency was then assessed by enumerating the resulting transformant colonies.

After performing the CTCNT-P procedure, numerous colonies appeared on minimal medium (MM) plates supplemented with 50 µg/mL kanamycin and 40 µg/mL tryptophan. In contrast, no colonies were observed when either the donor or recipient strain was processed individually and plated under selective antibiotic pressure, indicating that the colony formation was specifically due to plasmid transfer and not spontaneous mutations. Transformants were verified for plasmid presence using PCR (Figure S1).

To further confirm that plasmid transfer was mediated by natural transformation, we tested a recipient strain in which *comK*, the master regulator of competence, was deleted. No colonies emerged on the selective plates when this mutant strain was co-cultured with the donor strain (Figure 1B), verifying that natural transformation was responsible for the observed plasmid transfer.

We then validated the origin of the transformants by streaking them onto trypan blue starch plates (Figure S2). The donor strain (TLPHMC-G), with its disrupted *amyE* gene, cannot produce hydrolytic zones on these plates. Therefore, the appearance of hydrolysis zones indicates that the transformants originated from *B. subtilis* 168, which had acquired the pBE2 plasmid from the donor strain. As shown in Supplementary Figure S1, all tested transformants displayed hydrolytic rings and were confirmed to harbor the pBE2 plasmid, thereby establishing their derivation from *B. subtilis* 168.

### 2.2. Optimization of CTCNT-P in B. subtilis

To optimize the efficiency of plasmid transfer by CTCNT-P in *B. subtilis*, we first investigated the effect of the donor-to-recipient ratio. The donor strain (TLPHMC-G/pBE2) and the recipient strain (*B. subtilis* 168) were mixed at volume ratios ranging from 10:1 to 1:10 (Donor/Recipient). Transformants were obtained across all tested conditions; however, the highest number of transformants occurred at a 1:1 ratio. This ratio was therefore adopted for all subsequent experiments.

**Figure 2:**
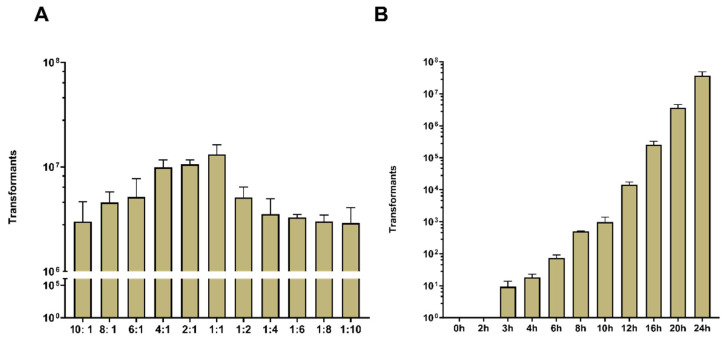
The ratio of donor and recipient and co-culture duration affect the number of transformants obtained by CTCNT-P. (**A**) Number of transformants obtained at different donor-to-recipient ratios: CTCNT-P was performed between TLPHMC-G/pBE2 (donor strain) and *B. subtilis* 168 (recipient strain) using minimal medium (MM) plates supplemented with 30 µg/mL kanamycin. The donor-to-recipient ratios were tested at 10:1, 8:1, 6:1, 4:1, 2:1, 1:1, 1:2, 1:4, 1:6, 1:8, and 1:10 (vol/vol), with a total mixture volume of 30 µL. Prior to mixing, the optical density (OD600) of donor and recipient cultures was adjusted to 1.5 and 1.3, respectively. Transformants were counted 36 h after incubation on selective plates. Data are presented as mean values ± standard deviation (SD), with error bars representing the SD (*n* = 3). (**B**) Influence of co-culture duration on the number of transformants via CTCNT-P: Donor and recipient strains were mixed at a 1:1 ratio and incubated for various durations: 0 h, 2 h, 3 h, 4 h, 6 h, 8 h, 10 h, 12 h, 16 h, 20 h, and 24 h. After incubation, the filter membrane was carefully removed using sterile tweezers, transferred into a tube, washed with 1 mL PBS (pH 7.2–7.4), diluted in appropriate gradients, and plated onto selective plates. Data are presented as mean values ± SD, with error bars representing the SD (*n* = 3).

Next, we examined the effect of co-culturing duration on transformation efficiency. Donor and recipient strains were co-cultured on a filter membrane placed on selective plates for varying durations, as outlined in Figure 1A. After incubation, the filter membrane was washed with PBS, and the surviving cells were plated onto a selective medium. Transformants began to appear 3 h after co-culture initiation, with the number of transformants gradually increasing over time (Figure 2B). A 12-h incubation time was identified as optimal, as it produced sufficient transformants for accurate quantification without oversaturation.

### 2.3. CTCNT Is Significantly More Efficient than DTCNT for Plasmid Transfer

To compare the efficiency of direct transformation (DTCNT-P) and cell-to-cell natural transformation (CTCNT-P), we tested two sources of transforming DNA: extracted plasmids and donor cells (TLPHMC-G) containing the plasmids. We used two plasmids—pGK12H (4.2 kb, chloramphenicol-resistant) and pGK12H-K (5.6 kb, kanamycin-resistant)—and quantified their copy numbers at approximately 5 per cell using quantitative PCR (qPCR) [23].

The concentration of plasmid DNA in donor cell cultures was estimated to be approximately 2.86 × 10⁻^3^ ng/mL, using the formula:

(1) $$Plasmid\;concentration=\frac{Cell\;density\times Plasmid\;copy\;number\times DNA\;size}{Avogadro’s\;number}$$

where cell density = 1.15 × 10^8^ cells/mL, plasmid copy number = 5, and Avogadro’s number ≈ 6.02 × 10^23^.

**Figure 3:**
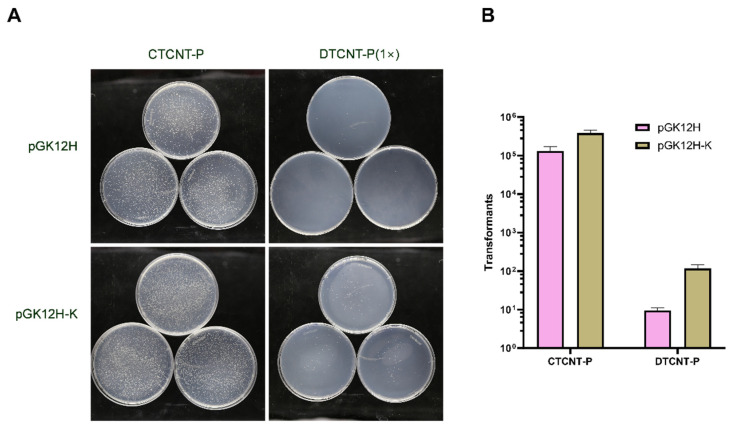
CTCNT-P is much more efficient than DTCNT-P. (**A**) Representative plates to (**B**) the number of transformants for CTCNT-P and DTCNT-P: *B. subtilis* 168 was used as the recipient strain, while purified plasmids pGK12H and pGK12H-K served as the transforming DNA for DTCNT-P. For CTCNT-P, the donor strain TLPHMC-G carrying either pGK12H or pGK12H-K was used. In the CTCNT-P assays, purified plasmids were used at an equivalent concentration, with pGK12H estimated to be 2.86 × 10⁻^3^ ng/mL based on the Formula (1). Here, the cell density is 1.15 × 10⁸ cells/mL, plasmid copy number is ~5, DNA size per base pair is 660 Da, and Avogadro’s number (NA) is 6.02 × 10^23^. Data are presented as mean values ± standard deviation (SD), with error bars representing the SD (*n* = 3).

As shown in Figure 3, the standard CTCNT-P procedure resulted in 10⁵ to 10^6^ transformants on selective plates. In contrast, when an equivalent amount of purified plasmid DNA was used in DTCNT-P, only 10^1^ to 10^2^ transformants were obtained. These results demonstrate that CTCNT is 10^3^ to 10^4^ times more efficient than DTCNT for plasmid transfer under identical experimental conditions.

The substantial efficiency of CTCNT-P suggests that natural plasmid transfer involves cell contact or other physiological processes that significantly enhance DNA uptake and transformation efficiency as compared to the direct introduction of purified plasmid DNA.

Given the high efficiency of CTCNT-P, we hypothesized that it could facilitate genetic manipulation if the plasmid contains homologous sequences to chromosomal DNA. To test this, we targeted the tryptophan auxotrophy of *B. subtilis* 168, which results from a disruption in the *trpC* gene.

We cloned the *trpC* gene from strain RO-NN-1 [31] into plasmids pGK12H and pGK12H-K, placing it under the control of the P43 promoter. This resulted in the plasmids pGK12H-*trpC* and pGK12H-K-*trpC*, which were then introduced into the donor strain TLPHMC-G for the CTCNT-P assay.

**Figure 4:**
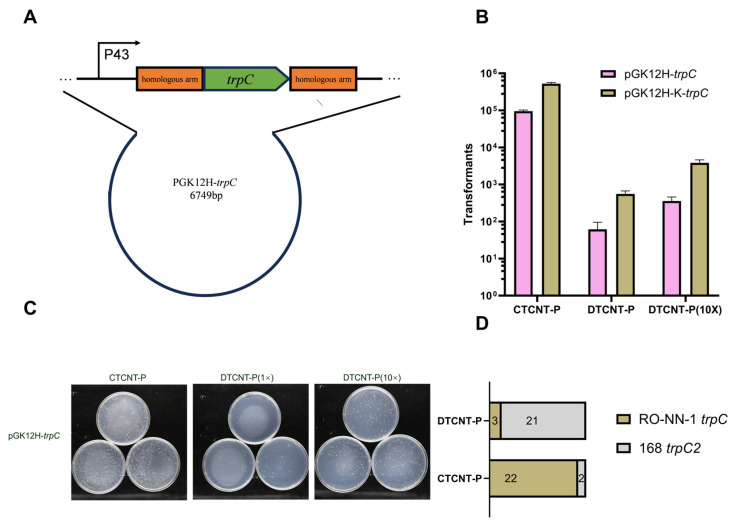
CTCNT-P can promote homologous recombination in the presence of homologous arms in the plasmid. (**A**) Map of pGK12H-*trpC*: The *trpC* gene from strain RO-NN-1 was cloned into plasmids pGK12H and pGK12H-K under the control of the P43 promoter. Both constructs, pGK12H-*trpC* and pGK12H-K-*trpC*, included 500 bp homologous arms upstream and downstream of the *trpC* gene to facilitate homologous recombination. (**B**) Number of transformants obtained by CTCNT-P and DTCNT-P: Transformants for pGK12H-*trpC* and pGK12H-K-*trpC* were selected on minimal medium (MM). In the DTCNT-P group, 10 times the plasmid concentration present in the donor cells (2.86 × 10^−2^ ng/mL) was used as the DNA source. (**C**) Representative plate images: shows representative plate images for CTCNT-P and DTCNT-P using TLPHMC-G/pGK12H-*trpC* as the donor and *B. subtilis* 168 as the recipient strain. (**D**) Recombination events among transformants: The numbers of recombinants were analyzed among transformants obtained by CTCNT-P and DTCNT-P. Six transformants were randomly selected from each group, for a total of 48 transformants, and the *trpC* loci in their chromosomes were amplified and sequenced to confirm homologous recombination events. Results of the analysis are presented in Figure S3. Data are presented as mean values ± standard deviation (SD), with error bars representing the SD (*n* = 3).

Tryptophan-positive (trp⁺) transformants were selected on a minimal medium (MM). Due to the low efficiency of DTCNT-P, we tested the same or tenfold higher concentrations of purified plasmid DNA. As shown in Figure 4A,B, CTCNT-P produced 10^6^ to 10^7^ transformants, whereas DTCNT-P yielded only 10^1^ to 10^2^ transformants under identical conditions.

To confirm whether the *trpC* mutation correction occurred via homologous recombination, we sequenced the *trpC* loci of 24 transformants. Remarkably, 22 of 24 transformants (91.7%) obtained from CTCNT-P resulted from homologous recombination, replacing the mutated *trpC* on the chromosome (Figure 4D and Figure S3). In contrast, only 3 of 24 transformants (12.5%) obtained from DTCNT-P exhibited homologous recombination.

These results highlight the superior efficiency of CTCNT-P, not only for plasmid transfer but also for facilitating homologous recombination when the plasmid contains homologous DNA sequences.

### 2.4. Antibiotic Stress Enhances Plasmid Transfer Efficiency via CTCNT-P in B. subtilis

CTCNT-mediated chromosomal DNA transfer occurs more efficiently when recipient cells are pre-cultured in a minimal medium (MM) compared to an LB medium. This improvement likely arises from MM’s role in enhancing competence development in recipient cells [28]. Additionally, exposing the donor-recipient cell mixture to appropriate antibiotic concentrations facilitates chromosomal DNA transfer via CTCNT. Here, we examined whether antibiotic stress could similarly enhance plasmid transfer efficiency by CTCNT.

For this experiment, the donor strain carried a plasmid encoding resistance to a specific antibiotic, while the recipient strain was sensitive to the antibiotic. Donor and recipient cells were co-cultured on a filter membrane placed on plates containing various antibiotic concentrations. After 12 h of incubation, the cells were washed off the membrane, diluted, and spread onto selective plates to quantify transformants.

**Figure 5:**
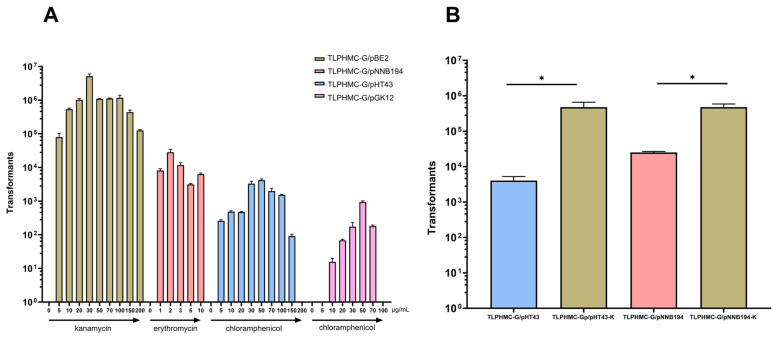
The presence of antibiotics during the co-culture period affects the efficiency of plasmid transfer by CTCNT. (**A**) The influence of antibiotic concentration on CTCNT efficiency: Donor strains (TLPHMC-G/pHT43, TLPHMC-G/pGK12, TLPHMC-G/pNNB194, or TLPHMC-G/pBE2) and the recipient strain (*B. subtilis* 168) were mixed in a 30 µL volume and spotted onto a filter membrane placed on minimal medium (MM) agar plates containing varying concentrations of the indicated antibiotics. After 12 h of co-culture at 37 °C, cells on the filter membrane were washed with PBS (pH 7.2–7.4) and plated onto selective plates containing the same antibiotic concentration. The X-axis represents the antibiotic concentration in the co-culture plates, while the Y-axis shows the number of transformants obtained. For donor strain TLPHMC-G/pBE2, co-culture plates were supplemented with kanamycin (K). For donor strain TLPHMC-G/pNNB194, co-culture plates contained erythromycin (E). For donor strains, TLPHMC-G/pHT43 and TLPHMC-G/pGK12, co-culture plates were supplemented with chloramphenicol (C). (**B**) Kanamycin significantly enhances plasmid transfer efficiency by CTCNT: To investigate the impact of kanamycin specifically, the cat cassette in pHT43 and the erm cassette in pNNB194 were replaced with the aph cassette, conferring resistance to kanamycin. The resulting plasmids (pHT43-K and pNNB194-K) were introduced into the donor strain TLPHMC-G, and CTCNT-P experiments were performed using equal amounts of donor and recipient cells. The number of transformants was quantified and plotted for comparison. Kanamycin-resistant plasmids showed significantly higher transformation efficiency compared to the original plasmids. Statistical analysis using a multiple comparison test revealed significant differences: TLPHMC-G/pHT43 vs. TLPHMC-G/pHT43-K: adjusted *p*-value = 0.0012 (t(8) = 5.491). TLPHMC-G/pNNB194 vs. TLPHMC-G/pNNB194-K: adjusted *p*-value = 0.0016 (t(8) = 5.237). Data are presented as mean values ± standard deviation (SD), with error bars representing the SD (*n* = 3). Statistical significance is denoted as * *p* < 0.05.

Our results demonstrated that the presence of antibiotics during co-culture was critical for plasmid transfer, and transformation efficiency varied with antibiotic concentration (Figure 5). For donor bacteria carrying chloramphenicol-resistant plasmids (pGK12 or pHT43), the highest number of transformants was observed at a chloramphenicol concentration of 50 μg/mL, yielding 9.34 × 10^2^ and 4.17 × 10^3^ transformants, respectively. For the erythromycin-resistant plasmid pNNB194, the optimal erythromycin concentration was 2 μg/mL, producing 2.82 × 10^4^ transformants.

When the donor strain harbored the kanamycin-resistant plasmid pBE2, the optimal kanamycin concentration was 30 μg/mL, resulting in the highest transformation efficiency, with 5.12 × 10^6^ transformants.

The three plasmids used in these experiments confer resistance to kanamycin, chloramphenicol, and erythromycin—antibiotics that inhibit protein translation. However, these antibiotics target the bacterial ribosome through distinct mechanisms: kanamycin binds to the 30S ribosomal subunit, interfering with the initiation complex and causing mRNA misreading, while chloramphenicol and erythromycin bind to the 50S ribosomal subunit to inhibit protein synthesis.

We hypothesized that the differences in transformation efficiency observed with these antibiotics could be due to their distinct effects on donor and recipient cells during the co-culture period on the filter membrane.

To further test the role of antibiotics in plasmid transfer efficiency, we repeated the experiments using kanamycin for the initial co-culture step, regardless of the resistance marker of the test plasmids. After co-culturing, transformants were selected on plates containing antibiotics corresponding to the resistance markers of the plasmids.

As shown in Figure S4B, the kanamycin-resistant plasmid pBE2 produced a large number of transformants. In contrast, the chloramphenicol-resistant plasmid pGK12H and the erythromycin-resistant plasmid pNNB194 resulted in 10^3^ to 10^4^ times fewer transformants. This outcome was significantly lower than the transformation efficiencies obtained with the standard procedure, where the same antibiotic was used in both the co-culture and selection steps.

These results suggest that the reduced transformation efficiency of pGK12H and pNNB194 is not caused by antibiotic differences during the initial co-culture step. Instead, they imply that kanamycin specifically enhances plasmid transfer by CTCNT through an unknown mechanism.

To confirm kanamycin’s role in facilitating plasmid transfer by CTCNT, we replaced the resistance markers in plasmids pHT43 and pNNB194 with the kanamycin resistance cassette from pBE2, generating pHT43-K and pNNB194-K. These modified plasmids were then used in the CTCNT-P assay under standard conditions, with TLPHMC-G as the donor strain.

Transformants were selected on minimal medium (MM) supplemented with tryptophan (40 µg/mL) and containing either chloramphenicol (50 µg/mL), kanamycin (30 µg/mL), or erythromycin (2 µg/mL).

As shown in Figure 5B, the number of transformants increased more than 100-fold when donor strains harboring pHT43-K or pNNB194-K were used, compared to their original counterparts (TLPHMC-G/pHT43 or TLPHMC-G/pNNB194). These results confirm that kanamycin is significantly more effective than chloramphenicol or erythromycin in promoting plasmid transfer via CTCNT. This observation is consistent with previous findings for chromosomal transfer [28]. To verify that the transformants resulted from the transfer of pBE2 from TLPHMC-G to *B. subtilis* 168, they were streaked onto trypan blue starch plates. Since the amyE gene is disrupted in TLPHMC-G, it cannot form hydrolysis zones on trypan blue starch plates. Consequently, if the transformants produced hydrolytic rings, they must have originated from *B. subtilis* 168, which had acquired the pBE2 plasmid from the donor strain. As shown in Supplementary Figure S1, all tested transformants exhibited hydrolytic zones and harbored the plasmid pBE2, confirming their derivation from *B. subtilis* 168. To optimize the transformation efficiency of CTCNT-P in *B. subtilis*, we first investigated whether the ratio of the donor strain (TLPHMC-G/pBE2) to the recipient strain (168) was critical for plasmid transfer. We mixed the two strains in volume ratios ranging from 10:1 to 1:10 (Donor/Recipient). While transformants were obtained at high frequencies across all tested conditions, the highest number of transformants was observed when the volume ratio of donor to recipient was 1:1. Consequently, this 1:1 ratio was adopted for all subsequent experiments.

### 2.5. The Efficiency of Plasmid Transfer via CTCNT Decreases with Increasing Plasmid Size

**Figure 6:**
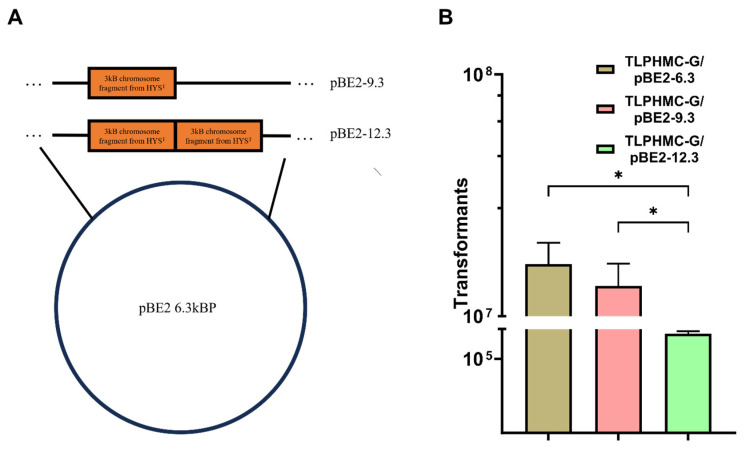
The size of the plasmid affects the efficiency of plasmid transfer by CTCNT-P in *B. subtilis*. (**A**) Map of plasmids pBE2, pBE2-9.3, and pBE2-12.3, which contain 3 kb and 6 kb DNA fragments cloned from HYS, respectively. (**B**) The donor strain TLPHMC-G carrying plasmids pBE2 (6.3 kb), pBE2-9.3, or pBE2-12.3 was used in CTCNT-P experiments. The results showed that the number of transformants decreased as plasmid size increased. In addition, the stability of the plasmids in transformants was evaluated (Figure S5). Statistical analysis using analysis of variance (ANOVA) revealed a significant effect of plasmid size on transformation efficiency (F(2, 6) = 11.01, *p* = 0.0098, R^2^ = 0.7859). Data are presented as mean values ± standard deviation (SD), with error bars representing the SD (*n* = 3). Statistical significance is denoted as * *p* < 0.05.

To determine whether plasmid size influences CTCNT-P efficiency, we constructed a series of plasmids of varying sizes using pBE2 (6.3 kb) as the backbone. A 3 kb or 6 kb DNA fragment from the chromosome of Pseudomonas donghuensis HYS [32] was inserted into pBE2, generating plasmids pBE2-9.3 (9.3 kb) and pBE2-12.3 (12.3 kb), respectively (Figure 6A).

These plasmids were transformed into the donor strain TLPHMC-G, and the resulting strains were used in CTCNT-P assays with *B. subtilis* 168 as the recipient strain.

As shown in Figure 6B, the number of transformants decreased significantly as plasmid size increased. Specifically, pBE2-9.3 and pBE2-12.3 produced far fewer transformants compared to the original pBE2 plasmid. These results demonstrate that the efficiency of plasmid transfer via CTCNT is negatively correlated with plasmid size.

### 2.6. Cell-to-Cell Natural Transformation Promotes Plasmid Transfer Between Different Bacilli Species

**Figure 7:**
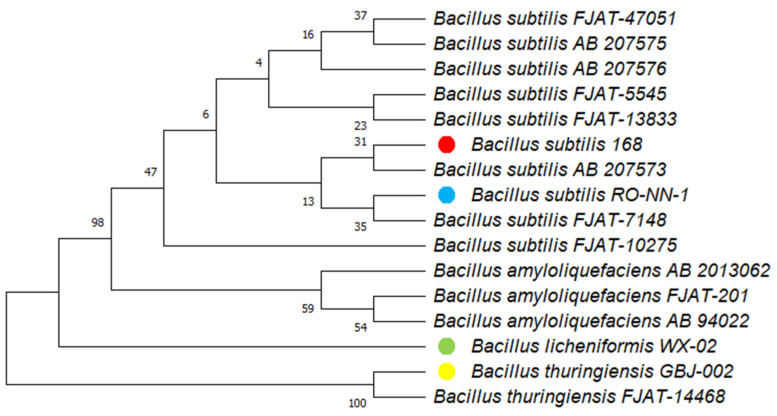
The phylogenetic relationship of 16 *Bacillus* strains based on 16S rDNA. The phylogenetic relationship of the 16 *Bacillus* strains used in this study was inferred using the Minimum Evolution (ME) method. The optimal phylogenetic tree is presented, with bootstrap values (from 1000 replicates) shown next to the branches, indicating the percentage of replicate trees in which the associated taxa clustered together. Evolutionary distances were calculated using the Jukes–Cantor method and are expressed as the number of base substitutions per site. The Close-Neighbor-Interchange (CNI) algorithm (search level 1) was employed to optimize the ME tree, while the Neighbor-Joining (NJ) algorithm was used to generate the initial tree. Ambiguous positions were removed for each sequence pair using the pairwise deletion option. The final dataset consisted of 1470 positions. All evolutionary analyses were performed using MEGA11. *Bacillus subtilis* 168 (red), *Bacillus subtilis* RO-NN-1 (blue), *Bacillus licheniformis* WX-02 (green), and *Bacillus thuringiensis* GBJ-002 (yellow) have been sequenced, and additional genome data can be found in the NCBI database.

Thus far, CTCNT-P experiments have focused on laboratory strains of *B. subtilis*. To evaluate the broader applicability of CTCNT-P, we tested its ability to transfer plasmids into environmental isolates of *B. subtilis* and other *Bacillus* species. The recipient bacteria included nine environmental isolates of *B. subtilis*, three strains of *B. amyloliquefaciens*, two strains of *B. thuringiensis*, and one strain of *B. licheniformis*. A phylogenetic tree based on their 16S rDNA sequences is shown in Figure 7. Antibiotic sensitivity profiling confirmed that all strains were sensitive to kanamycin, erythromycin, and chloramphenicol.

We conducted CTCNT-P experiments using three donor strains: TLPHMC-G/pBE2, TLPHMC-G/pNNB194, and TLPHMC-G/pGK12. For comparison, plasmid transfer efficiency was also evaluated using Spizizen’s transformation method and electroporation. The results are summarized in Table 1.

Using TLPHMC-G/pBE2 as the donor, the kanamycin-resistant plasmid pBE2 was successfully transferred into 10 out of 15 wild *Bacillus* strains, including seven isolates of *B. subtilis* and three isolates of *B. amyloliquefaciens*. In contrast, application of the Spizizen’s protocol failed to transform any environmental *Bacillus* strains, while electroporation yielded transformants in only two *B. subtilis* isolates (FJAT-5545 and FJAT-7148).

Using TLPHMC-G/pNNB194 as the donor, the plasmid conferring erythromycin resistance, pNNB194, was transferred into 10 out of 15 strains, including seven isolates of *B. subtilis*, two isolates of *B. amyloliquefaciens*, and one isolate of *B. thuringiensis*. Spizizen’s protocol resulted in transformants for only four strains, while electroporation achieved transfer in six strains (four *B. subtilis* and two *B. amyloliquefaciens*).

Using TLPHMC-G/pGK12 as the donor, the plasmid conferring chloramphenicol resistance, pGK12, was successfully transferred into eight *B. subtilis* isolates, three *B. amyloliquefaciens* strains, one *B. thuringiensis* strain, and one *B. licheniformis* strain. In contrast, Spizizen’s method achieved transfer in only one strain, and electroporation failed to produce transformants for any strain.

PCR analysis confirmed the presence of the plasmids in all transformants (Supplementary Figure S6). Collectively, these results demonstrate that CTCNT-P is significantly more efficient than traditional methods like Spizizen transformation and electroporation for transferring plasmids into wild *Bacillus* strains.

## 3. Discussion

In this study, the CTCNT-P process was characterized in *Bacillus subtilis* and other *Bacillus* species. The results revealed that plasmid transfer efficiency is strongly influenced by factors such as the donor-to-recipient cell ratio, co-culture duration, and exposure to stress. Additionally, CTCNT-P demonstrated significantly higher efficiency compared to traditional plasmid transfer methods, including electroporation [25] and the Spizizen transformation method [26]. Significantly, this method has been successfully applied to environmental isolates of *B. subtilis* and other *Bacillus* species, including *B. amyloliquefaciens* and *B. licheniformis*. Some of these strains are known to be challenging for plasmid introduction using conventional methods. These findings position CTCNT-P as a simple, efficient, and versatile method for plasmid transfer across a wide range of *Bacillus* strains. This approach holds particular promise for modifying wild *Bacillus* strains that are traditionally difficult to transform with DNA, thereby opening new possibilities in agricultural and industrial applications.

The genetic manipulation of wild-type *Bacillus* species has been a focal point in biotechnology, given their importance in industrial applications and their potential for producing valuable metabolites. Among the methods used for genetic manipulation, plasmid transfer is pivotal for introducing new traits or modifying existing ones. In our previous studies [28,29], we discovered the phenomenon of cell-to-cell natural transformation (CTCNT) in *B. subtilis*, which facilitates the transfer of both chromosomal and plasmid DNA. We demonstrated that CTCNT can mediate the transfer of long continuous chromosomal DNA regions up to 347 kb, and a recipient cell can receive up to 66 transferred DNA fragments. This underscores the importance of natural transformation in horizontal gene transfer (HGT) and in shaping the genomic landscape of *Bacillus* strains. In this study, we characterized the process of plasmid transfer by CTCNT, establishing a standard procedure and optimizing conditions for achieving the highest transformation efficiency. Interestingly, we found that using kanamycin as the selection marker can significantly increase transformation efficiency through an unknown mechanism. It is worth noting that the three resistances we tested are all associated with protein translation. Among them, kanamycin binds to the 30S subunit, interfering with the initiation complex and causing mRNA misreading. Chloramphenicol and erythromycin, on the other hand, bind to the 50S ribosomal subunit and inhibit protein synthesis. We currently do not know if there are other antibiotics that might also facilitate the occurrence of CTCNT. Considering that the transformation of exogenous DNA into bacterial cells can be influenced by the cell surface structure, it is reasonable to speculate that antibiotics that affect bacterial cell wall synthesis, such as ampicillin, might also enhance the efficiency of CTCNT. However, to date, there is no available ampicillin resistance gene that has been shown to be expressible in Bacillus species. Consequently, we have not conducted any tests in this regard.

Moreover, we found that CTCNT-P enables plasmid transfer in natural isolates of *B. subtilis* strains and other *Bacillus* species, which are challenging to transform using traditional approaches such as the Spizizen transformation method or electroporation. Studies have demonstrated that large plasmids can be transferred by modifying and enhancing the K-state of recipient bacteria [33].

For wild-type *Bacillus* species, the CTCNT-P method offers a simple and effective approach for plasmid transfer, even in the absence of a well-defined genetic background. By relying solely on the co-culture of donor and recipient strains under stress conditions (e.g., antibiotic stress or amino acid deprivation)—common in natural environments—this specialized form of natural transformation may be more prevalent than previously understood. As such, it represents a significant pathway for horizontal gene transfer (HGT) in microbial ecosystems. Given the significant potential of *Bacillus* species in synthetic biology and agriculture in the one-health era, CTCNT-P offers a simple and new feasible method for genetic modification of both laboratory and naturally isolated *Bacillus* strains. For our study, we used the TLPHMC-G strain as the universal donor, which provided the plasmid source. This strain, derived from a lab-model strain, was convenient to culture and allowed easy control over plasmid transfer due to its multiple auxotrophic defects. This made the culture process both convenient and cost-effective. However, because the genetic backgrounds of the donor and recipient strains are similar, there is a risk of non-selective chromosomal transfer. Therefore, using the CTCNT-P method to transfer plasmids to *Bacillus* strains with lower chromosomal similarity may help ensure the recipient strain maintains chromosomal consistency. Additionally, given the ubiquity of natural genetic transformation in bacteria, it is tempting to assume that CTCNT and CTCNT-P may be applicable to a wider range of bacterial species outside the *Bacilli* group, particularly those in which it is currently difficult to introduce foreign DNA using conventional laboratory methods.

While simply co-culturing donor and recipient strains under stress conditions can facilitate plasmid DNA transfer in *B. subtilis*, many factors can also influence transformation efficiency. We found that the duration of the co-culture period on the filter membrane (on the first selective plate) has the largest impact on transformation efficiency. Transformants could be obtained after the mixture was co-cultured on the filter membrane for just 3 h, indicating rapid plasmid transfer during the CTCNT-P assay. The number of transformants increased slowly during the first 4 h and then rapidly increased with prolonged incubation time, likely due to the quick replication of initially transformed cells. In addition to incubation time, the ratio of donor to recipient cells influenced the number of transformants. The highest number of transformants was achieved when the ratio of donor to recipient strain was 1:1, maximizing the contact probability between donor and recipient cells, as we previously demonstrated that CTCNT-P is a coordinated process requiring proximity. Antibiotic treatment during the co-culture period is critical for the success of CTCNT-P. In the absence of antibiotics, no transformants were obtained for any tested plasmids (Figure 5A), indicating that stress from antibiotics helps the CTCNT-P process. Which may cause cell death in the donor strains, resulting in the release of plasmids that facilitate transfer during CTCNT-P. Since the donor cells harboring the plasmids are resistant to the antibiotics on the selective plates, the effect of the antibiotics likely targets the recipient cells, which are sensitive to the antibiotics. The mechanism by which antibiotic stress during the co-culture period increases transformation efficiency is currently unknown, but it may stimulate the development of competence in recipient cells or alter their physiology, enhancing plasmid uptake. Notably, different antibiotics vary significantly in their effects; kanamycin appears to have the greatest impact on transformation efficiency compared to chloramphenicol and erythromycin. Chromosomal DNA transfer by CTCNT also observed similar effects of stress, though the roles of antibiotics differ depending on whether the donor or recipient cells are targeted.

CTCNT exhibits relative resistance to DNase I, suggesting that transferred DNA molecules (both chromosomal and plasmid DNA) are shielded from DNase I. One possibility is that CTCNT occurs through membranous connections between *B. subtilis* cells, similar to bacterial nanotubes [9,30,34]. However, while unbound plasmids can be transferred via nanotube-like structures, chromosomal DNA cannot. Thus, the mechanism of DNA transfer in CTCNT remains elucidated. Research on kin discrimination supports the hypothesis that closely related *Bacillus* strains exhibit a higher propensity for horizontal gene transfer. This observation underscores the significance of genetic relatedness in facilitating gene exchange among bacterial populations [35,36,37,38]. By co-cultivating mixed donor and recipient cells on a filter membrane and allowing them to grow for 12 h, we successfully facilitated the transfer of plasmids from laboratory *B. subtilis* strains to environmental isolates of *Bacillus* species (Table 1). Compared to the Spizizen transformation and electro-transformation techniques, our method demonstrated significantly improved success in transferring plasmids to a greater number of wild strains. This suggests that CTCNT could be employed to modify *Bacillus* strains that are difficult to transform by traditional approaches. Given the biotechnological potential of *Bacillus* species as biocontrol agents and cell factories for enzyme and high-value product production, CTCNT may be an effective method for engineering *Bacillus* strains for industrial applications.

## 4. Materials and Methods

### 4.1. Bacterial Growth Conditions

The bacterial strains and plasmids used in this study are listed in Supplementary Tables S1 and S2. All bacteria were cultured at 37 °C unless stated otherwise. *Escherichia coli* strains were grown in Lysogeny Broth (LB) or on LB agar plates (1.5% agar). *Bacillus subtilis* strains were cultured in LB or minimal medium (MM) containing 1.5% agar for solid media. When required, the following amino acids were added to MM at the specified concentrations: Tryptophan (Trp): 20 µg/mL, Lysine (Lys): 50 µg/mL, Methionine (Met): 50 µg/mL. The following antibiotics were supplemented for plasmid maintenance or selection: Kanamycin: 30 µg/mL (LB) or 50 µg/mL (MM), Ampicillin: 100 µg/mL, Erythromycin: 2 µg/mL, Chloramphenicol: 7.5 µg/mL (LB) or 50 µg/mL (MM). The amino acids and antibiotic reagents were purchased from Sangon Biotech (Shanghai, China). Antibiotics were added at varying final concentrations as indicated to evaluate their effects on CTCNT-P transformation efficiency.

### 4.2. Construction of Plasmids

Four *E. coli*–*B. subtilis* shuttle vectors were used in this study: pBE2 [39], pGK12H [29,40], pHT43 [41], and pNNB194 [42]. To test the effect of plasmid size on CTCNT-P efficiency, we constructed derivatives of pBE2: pBE2-9.3 (9.3 kb): A 3 kb DNA fragment was inserted into pBE2. pBE2-12.3 (12.3 kb): A 6 kb DNA fragment was added to pBE2-9.3. For testing kanamycin’s effect on transformation, the kanamycin-resistant cassette from pBE2 was cloned into pHT43 and pNNB194, generating pHT43-K and pNNB194-K. To test homologous recombination, the *trpC* gene was cloned from strain RO-NN-1 under the P43 promoter into pGK12H and pGK12H-K, resulting in pGK12H-*trpC* and pGK12H-K-*trpC*, respectively.

### 4.3. Preparation of Plasmid DNA

Plasmid DNA was purified from overnight 5 mL cultures of *Bacillus subtilis* using the TIANprep mini plasmid kit (Tiangen, Beijing, China) following the manufacturer’s protocol. The concentration of purified plasmid DNA was estimated using the formula:

(2) $$Plasmid\;concentration=\frac{Cell\;density\times Plasmid\;copy\;number\times DNA\;size}{Avogadro’s\;number}$$

Plasmid DNA was stored at −20 °C for future use.

### 4.4. Cell-to-Cell Natural Transformation Assay

The CTCNT-P assay was performed as described previously [28,29], with slight modifications.

Preparation of Donor and Recipient Strains:

The donor strain (TLPHMC-G harboring a plasmid) was grown overnight in LB supplemented with antibiotics. The recipient strain (e.g., *B. subtilis* 168 or wild isolates) was cultured overnight in MM supplemented with appropriate amino acids.

Co-Culture Process:

Donor and recipient cultures were diluted 1:100 into fresh LB and MM, respectively, and incubated at 37 °C until reaching a density of approximately 1 × 10⁸ cells/mL. Equal volumes (100 µL) of donor and recipient cultures were mixed and spotted onto a filter membrane (0.22 μm GSWP Mixed Cellulose Ester Membrane from Millipore, Merck KGaA, Darmstadt, Germany) placed on selective MM plates containing antibiotics.

Selection and Recovery:

After incubation for the specified duration (e.g., 12 h), the filter membrane was washed with PBS (pH 7.2–7.4) to collect cells. Cells were serially diluted and plated onto selective MM plates to count transformants.

### 4.5. Spizizen Transformation and Electroporation

The classical two-step protocol [43] was followed for the natural transformation of *Bacillus subtilis*. Electroporation was performed using an electroporator from Bio-Rad, Hercules, CA, USA, set to 25 µF, 200 Ω, and 2.1 kV [44].

### 4.6. Sequencing Methods and Statistical Analysis

Chromosomal loci of transformants were verified via PCR amplification and Sanger sequencing (Sangon Biotech, Shanghai, China). Sequence alignment was performed using SnapGene v 7.1.2 and DNAMAN software v10.0. 16S rDNA sequences of the *Bacillus* strains were amplified with primers 27-F and 1492-R. Statistical analysis was conducted using GraphPad Prism v10.1.2 (324). Comparisons were performed using *t*-tests or ANOVA, as appropriate.

### 4.7. Phylogenetic Analysis

The evolutionary relationships of 16 *Bacillus* strains were analyzed using MEGA11. The Minimum Evolution method was applied to infer the phylogenetic tree based on 16S rDNA sequences. Bootstrapping with 1000 replicates was used to assess tree reliability.

### 4.8. Data Available and Access Number

The 16S rDNA sequence and *trpC* sequence of 48 transformants in Figure 4 and FASTA data in Figure S3 can be found at https://doi.org/10.6084/m9.figshare.26780239.v1, dataset posted on 19August 2024. Details regarding the bacterial strains, plasmids, and primer sequences used in the experiments are provided in the supplementary materials, presented in tabular format.

## Figures and Tables

**Table 1:** Comparison of plasmid transfer in *B. subtilis* 168 and 15 wild *Bacillus* strains by CTCNT or classical transformation approaches.

Recipient			Donor								
Species	Number	Strain Name	TLPHMC-G/pBE2			TLPHMC-G/pNNB194			TLPHMC-G/pGK12		
		METHOD	CTCNT	SPIZIZEN	ELECTRO	CTCNT	SPIZIZEN	ELECTRO	CTCNT	SPIZIZEN	ELECTRO
*Bacillus subtilis*	1	168	+	+	+	+	+	+	+	+	+
	2	RO-NN-1	+	-	-	+	+	-	+	-	-
	3	FJAT-5545	+	-	+	+	+	+	+	-	-
	4	FJAT-7148	+	-	+	+	-	-	-	-	-
	5	FJAT-10275	-	-	-	+	+	-	+	+	-
	6	FJAT-13833	+	-	-	+	-	-	+	-	-
	7	FJAT-47051	-	-	-	-	-	+	+	-	-
	8	AB-207573	+	-	-	+	-	+	+	-	-
	9	AB-207575	+	-	-	-	-	+	+	-	-
	10	AB-207576	+	-	-	+	-	-	+	-	-
*Bacillus amyloliquefaciens*	11	FJAT-201	+	-	-	+	+	-	+	-	-
	12	AB-94022	+	-	-	+	-	+	+	-	-
	13	AB-2013062	+	-	-	-	-	+	+	-	-
*Bacillus thuringiensis*	14	FJAT-14468	-	-	-	+	-	-	-	-	-
	15	GBJ-002	-	-	-	-	-	-	+	-	-
*Bacillus licheniformis*	16	WX-02	-	-	-	-	-	-	+	-	-

NOTE: “+”: transformants were observed and passed the PCR test. (Figure S4). “-”: either transformants were not observed or transformants did not pass PCR test. SPIZIZEN: two-step Spizizen transfer method. ELECTRO: electroporation-transformation.

## Data Availability

Data are contained within the article.

## Supplementary Materials

The following supporting information can be downloaded at: https://www.mdpi.com/article/10.3390/ijms26020621/s1. Refs. [29,31,32,39,40,41,42,45,46,47,48] are cited in Supplementary Materials file.
