# Supplementary material for: Cell-to-Cell Natural Transformation Mediated Efficient Plasmid Transfer Between Bacillus Species
Source: Int J Mol Sci. 2025 Jan 13;26(2):621. doi: 10.3390/ijms26020621 (PMC11765539; doi:10.3390/ijms26020621)
Supplement: Supplementary file 1 [file ijms-26-00621-s001.zip › ijms-3408599-supplementary.pdf]

International Journal of *Molecular Sciences*

MDPI

Supplementary Material

Table S1. Strains used in this study

Strain	Genotype[i]	Source/reference
DH5*α*	*supE44 ΔlacU169 (φ80lacZΔM15) hsdR17 recA1 endA1 gyrA96 thi-1 relA1*	Lab stock
GM272	*dam dcm hsdS21 metB1 galK galT22 mtl-2 ton tsx supE44*	[1]
168	*trpC2*	[2]
168*ΔcomK*	*trpC2 ΔcomK*	[2]
RO-NN-1	Wild *bacillus subtilis* strain	[3]
HYS^T	*Pseudomonas donghuensis*	[4]
TLPHMC-G	*trpC2 ΔlysA ΔpheA ΔhisD ΔmetC ΔcysE amyE::Ppen-lacIΔ11-gfpmut2*	[5]
TLPHMC-G/pBE2	*trpC2 ΔlysA ΔpheA ΔhisD ΔmetC ΔcysE amyE::Ppen-lacIΔ11-gfpmut2* Km^R	[5]
TLPHMC-G/pBE2-9.3	*trpC2 ΔlysA ΔpheA ΔhisD ΔmetC ΔcysE amyE::Ppen-lacIΔ11-gfpmut2* add 3kB chromosome fragment from HYS^T to pBE2 Km^R	This work
TLPHMC-G/pBE2-12.3	*trpC2 ΔlysA ΔpheA ΔhisD ΔmetC ΔcysE amyE::Ppen-lacIΔ11-gfpmut2* add 6kB chromosome fragment from HYS^T to pBE2 Km^R	This work
TLPHMC-G/pBE2-*trpC*	*trpC2 ΔlysA ΔpheA ΔhisD ΔmetC ΔcysE amyE::Ppen-lacIΔ11-gfpmut2* express prototrophy *trpC* from RO-NN-1 Cm^R	This work
TLPHMC-G/pGK12H	*trpC2 ΔlysA ΔpheA ΔhisD ΔmetC ΔcysE amyE::Ppen-lacIΔ11-gfpmut2* Cm^R Erm^R	[5]
TLPHMC-G/pGK12H-K	*trpC2 ΔlysA ΔpheA ΔhisD ΔmetC ΔcysE amyE::Ppen-lacIΔ11-gfpmut2* Km^R	This work
TLPHMC-G/pGK12H-*trpC*	*trpC2 ΔlysA ΔpheA ΔhisD ΔmetC ΔcysE amyE::Ppen-lacIΔ11-gfpmut2* express prototrophy *trpC* from RO-NN-1 Cm^R Erm^R	This work
TLPHMC-G /pHT43	*trpC2 ΔlysA ΔpheA ΔhisD ΔmetC ΔcysE amyE::Ppen-lacIΔ11-gfpmut2* Cm^R	This work
TLPHMC-G /pHT43-K	*trpC2 ΔlysA ΔpheA ΔhisD ΔmetC ΔcysE amyE::Ppen-lacIΔ11-gfpmut2* Km^R	This work
TLPHMC-G /pNNB194	*trpC2 ΔlysA ΔpheA ΔhisD ΔmetC ΔcysE amyE::Ppen-lacIΔ11-gfpmut2* Erm^R	This work
TLPHMC-G /pNNB194-K	*trpC2 ΔlysA ΔpheA ΔhisD ΔmetC ΔcysE amyE::Ppen-lacIΔ11-gfpmut2* Km^R	This work
TLPHMC-G/pBE2	*trpC2* Km^R	This work
TLPHMC-G*ΔcomK*/pBE2	*trpC2* Km^R *comK* deleted	This work
TLPHMC-G/pGK12H	*trpC2* Cm^R	This work

TLPHMC-G /pNNB194	*trpC2* Erm^R	This work
FJAT-5545	Wild *bacillus subtilis* strain	Fujian Academy of Agricultural Sciences
FJAT-7148	Wild *bacillus subtilis* strain	Fujian Academy of Agricultural Sciences
FJAT-13833	Wild *bacillus subtilis* strain	Fujian Academy of Agricultural Sciences
FJAT-10275	Wild *bacillus subtilis* strain	Fujian Academy of Agricultural Sciences
FJAT-47051	Wild *bacillus subtilis* strain	Fujian Academy of Agricultural Sciences
AB-207573	Wild *bacillus subtilis* strain	CCTCC[ii]
AB-207575	Wild *bacillus subtilis* strain	CCTCC
AB-207576	Wild *bacillus subtilis* strain	CCTCC
FJAT-201	Wild *bacillus amyloliquefacients* strain	Fujian Academy of Agricultural Sciences
AB-94022	Wild *bacillus amyloliquefacients* strain	CCTCC
AB-2013062	Wild *bacillus amyloliquefacients* strain	CCTCC
FJAT-14468	Wild *bacillus thuringiensis* strain	Fujian Academy of Agricultural Sciences
GBJ-002	Nalidixic acid-resistant spontaneous mutant of 4Q7, *bacillus thuringiensis* subsp. *israelensis*	[6]
WX-02	*Bacillus licheniformis* strain	[7]

i Km^R, kanamycin resistance; Cm^R, chloramphenicol resistance; Erm^R, erythromycin resistance; Amp^R, ampicillin resistance.
ii CCTCC, China Center for Type Culture Collection.

Table S2. Plasmid used in this study.

Plasmid	Description	Source/reference
pBE2	*E. coli-B. subtilis* shuttle vector, Amp^R Km^R	[8]
pBE2-9.3	pBE2 joined with 3kb chromosome fragment from HYS^T to pBE2	This work
pBE2-12.3	pBE2 joined with 6kb chromosome fragment from HYS^T to pBE2	This work
pGK12H	E. coli-B. subtilis shuttle vector, temperature sensitive Cm^R Em^R, pGK12 derivative with multiple-cloning site from pUC18	[5,9]
pGK12H-K	pGK12H's Cm^R replaced by Km^R from pBE2	This work
pGK12H-*trpC*	pGK12H joined with prototrophy *trpC* from RO-NN-1	This work
pGK12H-K-*trpC*	pGK12H-K joined with prototrophy *trpC* from RO-NN-1	This work
pHT43	*E. coli-B. subtilis* shuttle vector, Amp^R Cm^R,	[10]
pHT43-K	pHT43's Cm^R replaced by Km^R from pBE2	This work
pNNB194	E. coli-B. subtilis shuttle vector, temperature sensitive Amp^R Erm^R	[11]
pNNB194-K	pNNB194's Erm^R replaced by Km^R from pBE2	This work

Table S3. Primers used in this study.

Primers	Sequence 5′ to 3′	description
pBE2-1	GGAATTGTGCTGTTTTATCCTT	Plasmid detection
pBE2-2	ACTCAGAAGGTTCGTCAACC	Plasmid detection
pGK12-1	CGGGCCATTTTGCGTAATAA	Plasmid detection
pGK12-2	CGTTTGTTGAACTAATGGGTG	Plasmid detection
pHT43-1	GCGAGGAAGCGGAAGA	Plasmid detection
pHT43-2	GCACCCATTAGTTCAACAAACG	Plasmid detection
pNNB194-1	TAGCAAACCCGTATTCCACG	Plasmid detection
pNNB194-2	AACCCTCACTAAAGGGAACA	Plasmid detection
27-F	AGAGTTTGATCCTGGCTCAG	16S rDNA amplify
1492-R	GGTTACCTTGTTACGACTT	16S rDNA amplify
trpC-testF	TCGTTTGCTTTCAGCAAAAA	*trpC* recombine test
trpC-testR	ATAAAAGCAGCAGTTCCGCT	*trpC* recombine test
pBE2-9.3-1	GATTTAGGTGACACTATAGAATACAAGCTTCGCGGATCCCCCGCTG	Cloning 3kb fragment from HYS^T to pBE2, HindIII
pBE2-9.3-2	AGCTCGGTACCCGGGGATCCTCTAGACGCGGATCCATGGATGAAAT	Cloning 3kb fragment from HYS^T to pBE2, XbaI
pBE2-12.3-1	AATTCGAGCTCGGTACCCGGGGATCCTCGCTGAGCACGACCATGTT	Cloning 3kb fragment from HYS^T to pBE2-6.3, BamHI
pBE2-12.3-1	ATATTTCCTTGCACGGCAAATCTAGACCATAACGATCAACGCGATC	Cloning 3kb fragment from HYS^T to pBE2-6.3, XbaI
pGK12H-K-1	GCCAAGCTTGCATGCCTGCAGGTCGACTGACACAGAAGAAGGCGATT	Cloning Km^R from pBE2 to pGK12H, SalI
pGK12H-K-2	AGCTCGGTACCCGGGGATCCTCTAGATCAAAATGGTATGCGTTTTG	Cloning Km^R from pBE2 to pGK12H, BamHI
pGK12H-*trpC*-1	TTGCATGCCTGCAGGTCGACTCTAGACATGCAGGCCGGGGCATATG	Cloning p43 promotor to pGK12H, XbaI
pGK12H-*trpC*-2	AGGTAAGAGAGGAATGTACACatgcttgaaaaaattatcaaacaaa	Overlapping primer of P43 and *trpC*
pGK12H-*trpC*-3	tttgtttgataattttttcaagcatGTGTACATTCCTCTCTTACCT	Cloning *trpC* from RO-NN-1 to pGK12H and

		pGK12H-K, overlapping primer of P43 and *trpC*
pGK12H-*trpC*-4	ACAGCTATGACCATGATTACGAATTCtcactccccaaacaaagctt	Cloning *trpC* from RO-NN-1 to pGK12H and pGK12H-K, EcoRI
pHT43-K-1	agcggaagagcgcccaatacgcatgcTTACGTTCAAAATGGTATGC	Cloning Km^R from pBE2 to Pht43, SphI
pHT43-K-2	tttaataaaattgatttagacaattgATGACGAAAAAGCCGATGAA	Cloning Km^R from pBE2 to Pht43, MfeI
pNNB194-K-1	ttaaaacttggaaattatcgtgatcaGATGACGAAAAAGCCGATGA	Cloning Km^R from pBE2 to pNNB194, BclI
pNNB194-K-2	actaaagggaacaaaagctggagctcTTACGTTCAAAATGGTATGC	Cloning Km^R from pBE2 to pNNB194, SacI

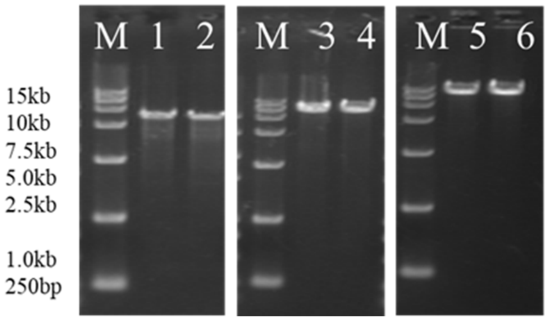

Figure S1. Conformation of the plasmid pBE2 identity in the transformants.

The transformants were continuously transferred on MM medium supplemented with kanamycin (50 μg/ml) for three passages. The plasmid was extracted from the transformants and digested with BamHI, followed by gel electrophoresis. M: DNA Marker Trans 2K Plus II; Lane 1: Plasmid isolated from transformant,pBE2(6.3 kb); Lane 2: pBE2 positive control; Lane 3: Plasmid isolated from transformant, pBE2-9.3; Lane 4: pBE2-9.3 positive control; Lane 5: Plasmid isolated from transformant pBE2-12.3; Lane 6: pBE2-12.3, positive control.

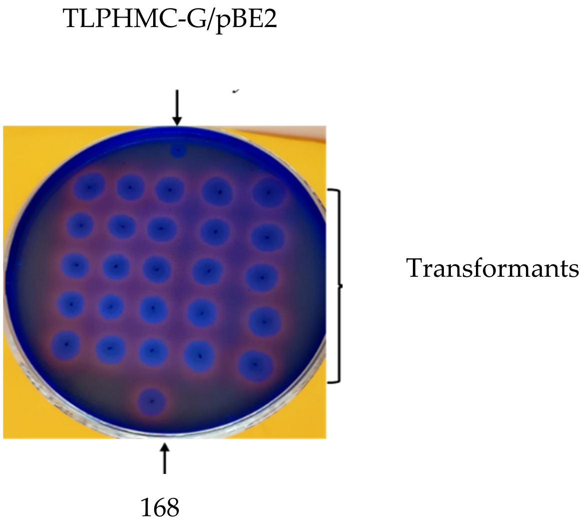

Figure S2. Identification of the origin of CTCNT-P transformants by Trypan blue starch plate test.

Using TLPHMC-G /PBE2 as the donor strain and *B. subtilis* 168 as the recipient strain, a CTCNT-P experiment was performed. Twenty-five transformants were randomly selected for analysis. These transformants were re-streaked onto LB plates containing 2% soluble starch and 0.001% trypan blue. Trypan blue, also known as Brilliant Blue G, has a strong affinity for large molecules such as starch but a weak affinity for smaller molecules. In the vicinity of amylase-producing bacterial colonies, large molecules like starch are hydrolyzed into smaller substances. As a result, the amount of starch around the colonies decreases, leading to a transparent zone around the colonies when stained with trypan blue. The donor strain TLPHMC-G, which lacks the *amyE* gene, is unable to hydrolyze starch and therefore does not produce a hydrolysis zone on the plate. However, the recipient strain 168 can hydrolyze starch. When recombinant strains are inoculated onto a trypan blue-starch plate, a hydrolysis zone is observed, indicating that plasmid transfer from TLPHMC-G to 168 has indeed occurred as intended.

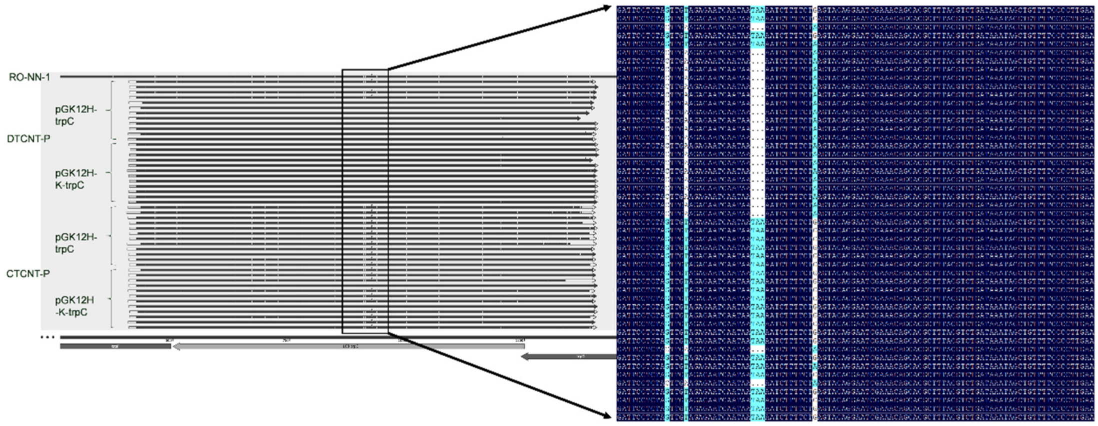

Figure S3. Sequencing results of the trpC gene in the transformants obtained by CTCNT-P or DTCNT-P between TLPHMC-G/pGK12H-trpC and B. subtilis 168.

The *trpC* locus was amplified from the transformants using the primer pair trpC-testF and trpC-testR and sequenced using Sanger sequencing. The sequencing results were analyzed with SnapGene and DNAman software. Twelve transformants were randomly selected from each of the four groups: 12 transformants from the DTCNT-P experiment with pGK12H-trpC and *B. subtilis* 168; 12 transformants from the DTCNT-P experiment with pGK12-K-trpC and *B. subtilis* 168; 12 transformants from the CTCNT-P experiment with TLPHMC-G/pGK12H-trpC and *B. subtilis* 168; and 12 transformants from the CTCNT-P experiment with TLPHMC-G/pGK12K-trpC and *B. subtilis* 168. In the DTCNT-P group, 3 out of 24 transformants had a *trpC* gene sequence identical to that of RO-NN-1, while in the CTCNT-P group, 21 out of 24 transformants had a *trpC* gene sequence identical to that of RO-NN-1.

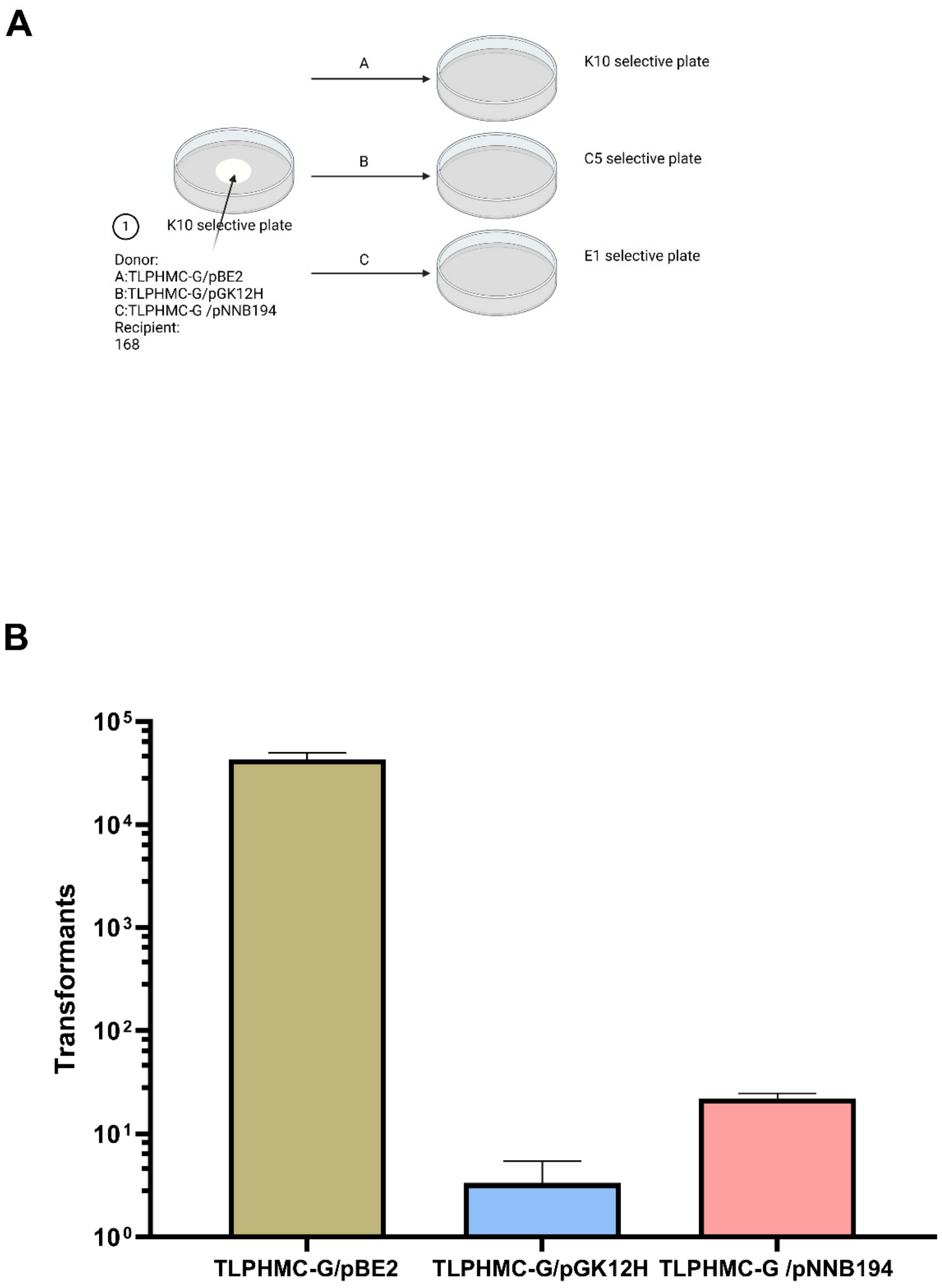

Figure S4. The influence of kanamycin on efficiency of plasmid transfer by CTCNT-P.

(A) An illustration of the experimental procedure. A mixture containing the indicated donor and recipient strains was spotted onto a filter membrane placed on plates containing kanamycin (10μg/mL). After co-incubation, the filter membrane was picked and washed. Cells were then harvested, diluted, and plated on selective plates containing antibiotics corresponding to the resistance markers of the tested plasmid. Kanamycin (10 μg/mL) selective plates were used for the TLPHMC-G/pBE2 group, chloramphenicol (5 μg/mL) selective plates were used for the TLPHMC-G/pGK12 group, and erythromycin (1 μg/mL) selective plates were used for the TLPHMC-G/pNNB194 group.

(B) Number of transformants from the three groups. Data are presented as mean values ± SD, with error bars representing the SD of the mean values (n = 3).

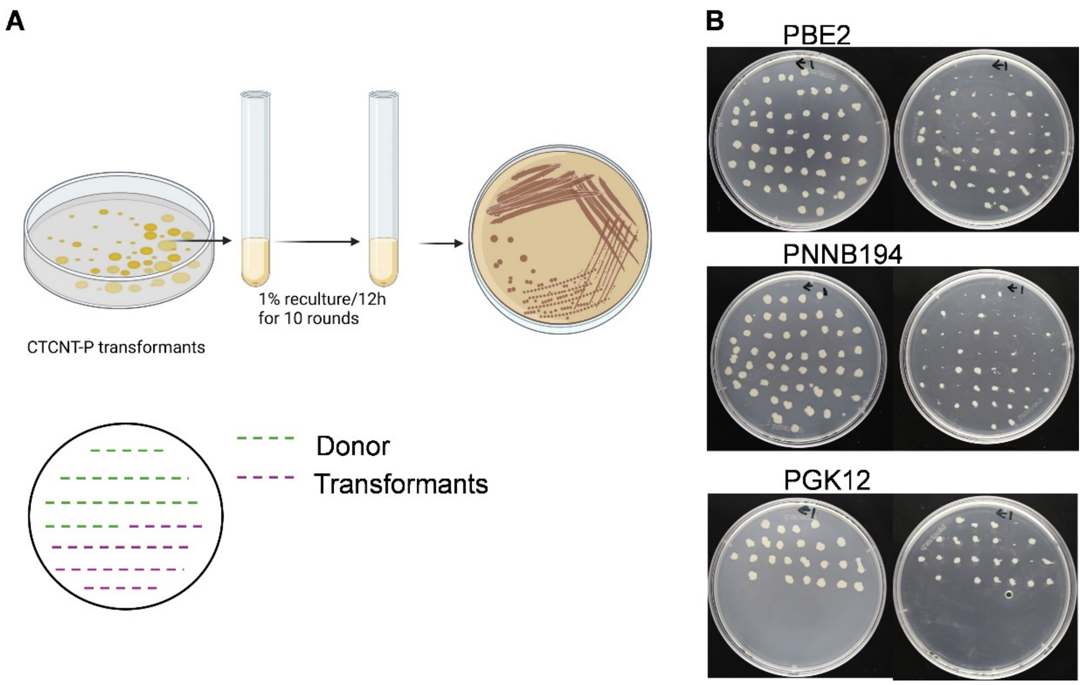

Figure S5. Plasmid stability test for CTCNT-P source transformants.

The donor bacteria and the transformants were continuously cultured for 10 rounds without resistance, and each round was cultured for 12 hours. The bacterial solution after 10 rounds was drawn on the non-resistant plate, and then single colonies were selected to verify the stability of the plasmid in the donor bacteria and the transformant. The resistant and non-resistant plates were seeded simultaneously.

(A) The flow chart of the non-resistant passaged strains and the schematic diagram of the donor and transformants seeded onto the plate. (B) The result of spot planting of single colonies selected after drawing lines of bacterial liquid after continuous passage to verify resistance. The left column is LB plate without resistance, and the right column is antibiotic plate with corresponding resistance. TLPHMC-G/pBE2 had 14/24 plasmid retention, and 168/pBE2 had 24/24 plasmid retention. TLPHMC-G/ pNNB194 had 9/24 retained plasmids, 168/pNNB194 had 19/24 plasmid retention, TLPHMC-G/pGK12H has 21/24 plasmids retention.

(A)

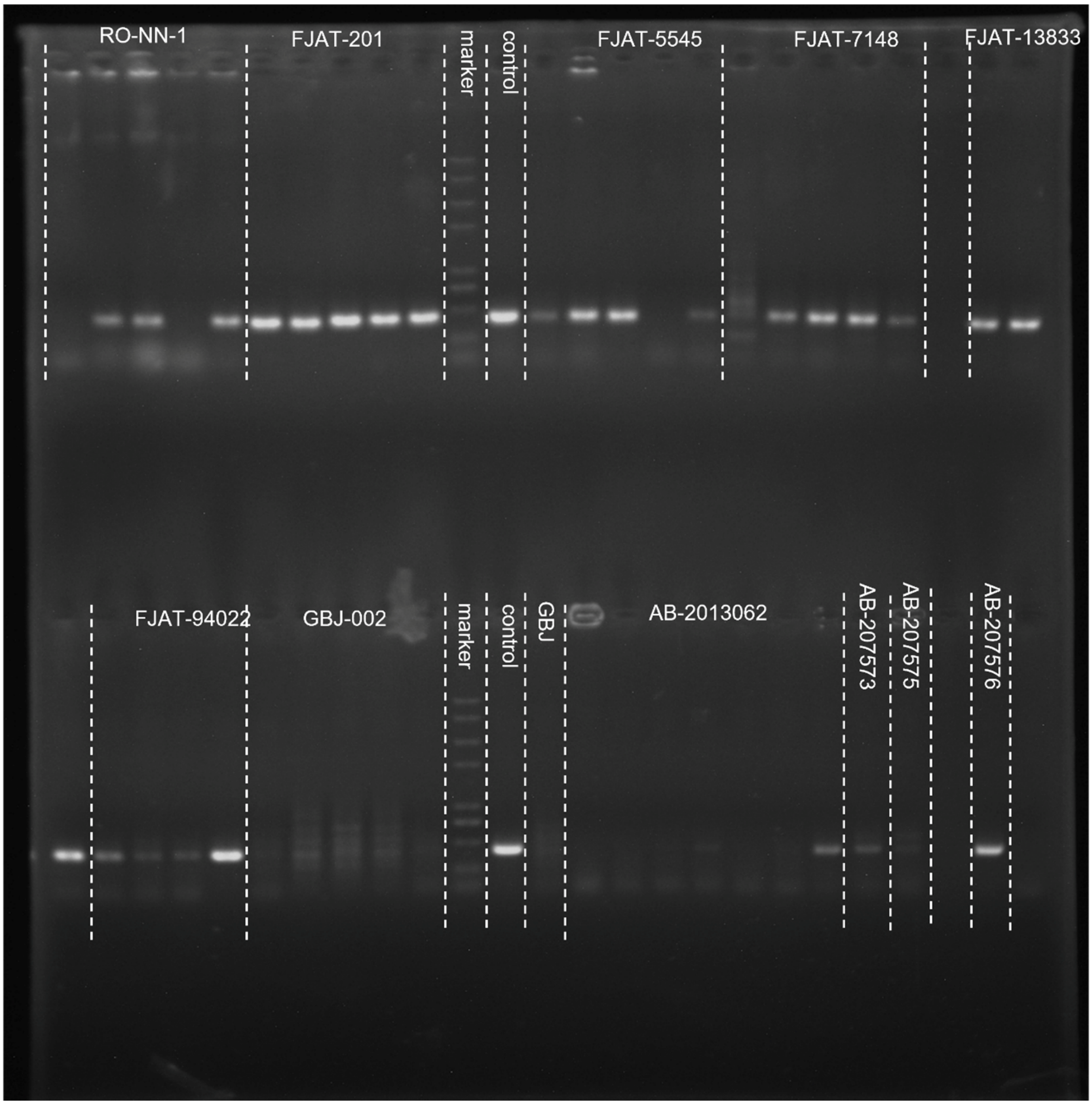
RO-NN-1
FJAT-201
marker
control
FJAT-5545
FJAT-7148
FJAT-13833
FJAT-94022
GBJ-002
marker
control
GBJ
AB-2013062
AB-207573
AB-207575
AB-207576

(B)

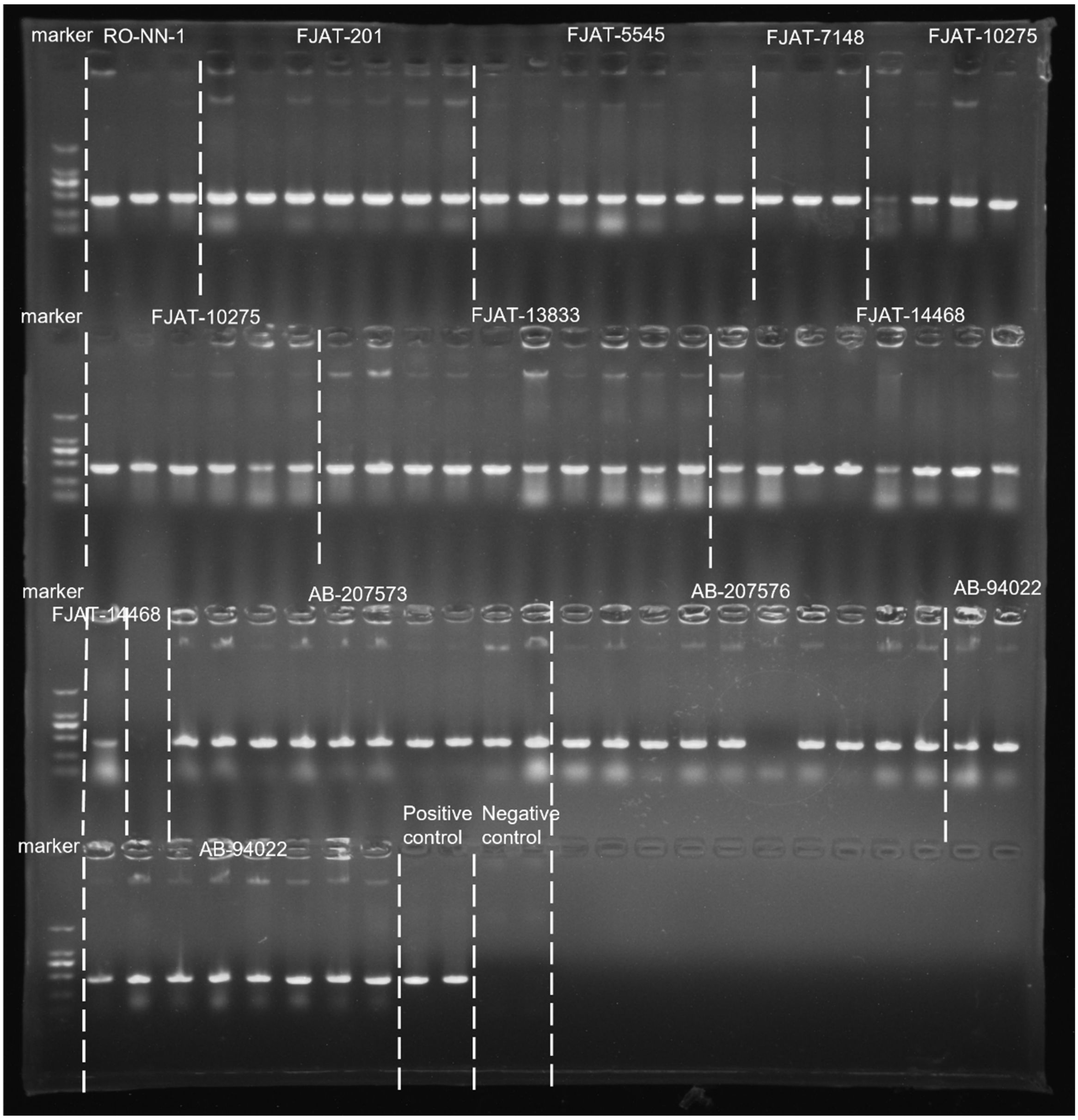
marker
RO-NN-1
FJAT-201
FJAT-5545
FJAT-7148
FJAT-10275
marker
FJAT-10275
FJAT-13833
FJAT-14468
marker
FJAT-14468
AB-207573
AB-207576
AB-94022
marker
AB-94022
Positive control
Negative control

(C)

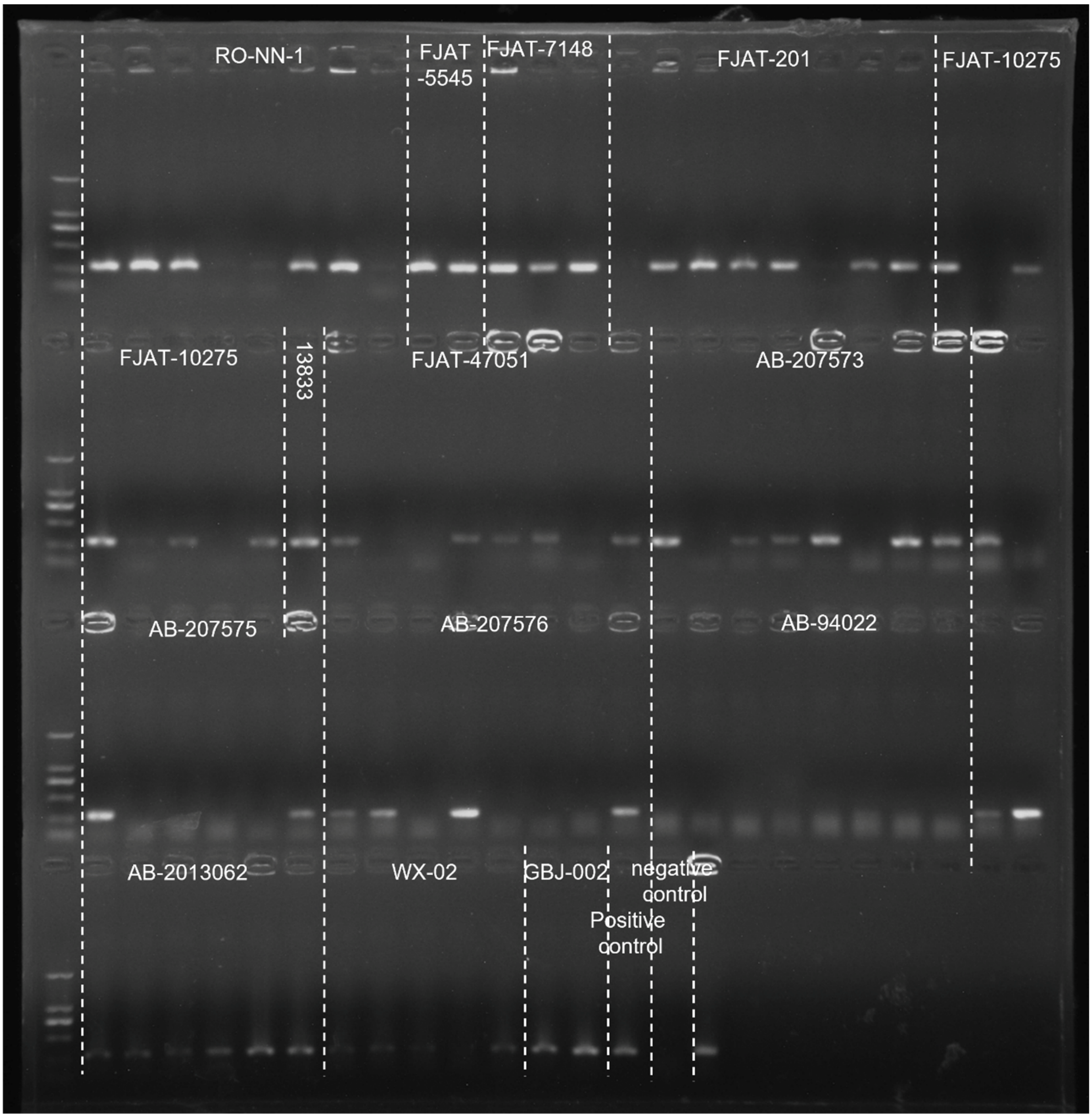
RO-NN-1
FJAT
-5545
FJAT-7148
FJAT-201
FJAT-10275
FJAT-10275
13833
FJAT-47051
AB-207573
AB-207575
AB-207576
AB-94022
AB-2013062
WX-02
GBJ-002
negative
control
Positive
control

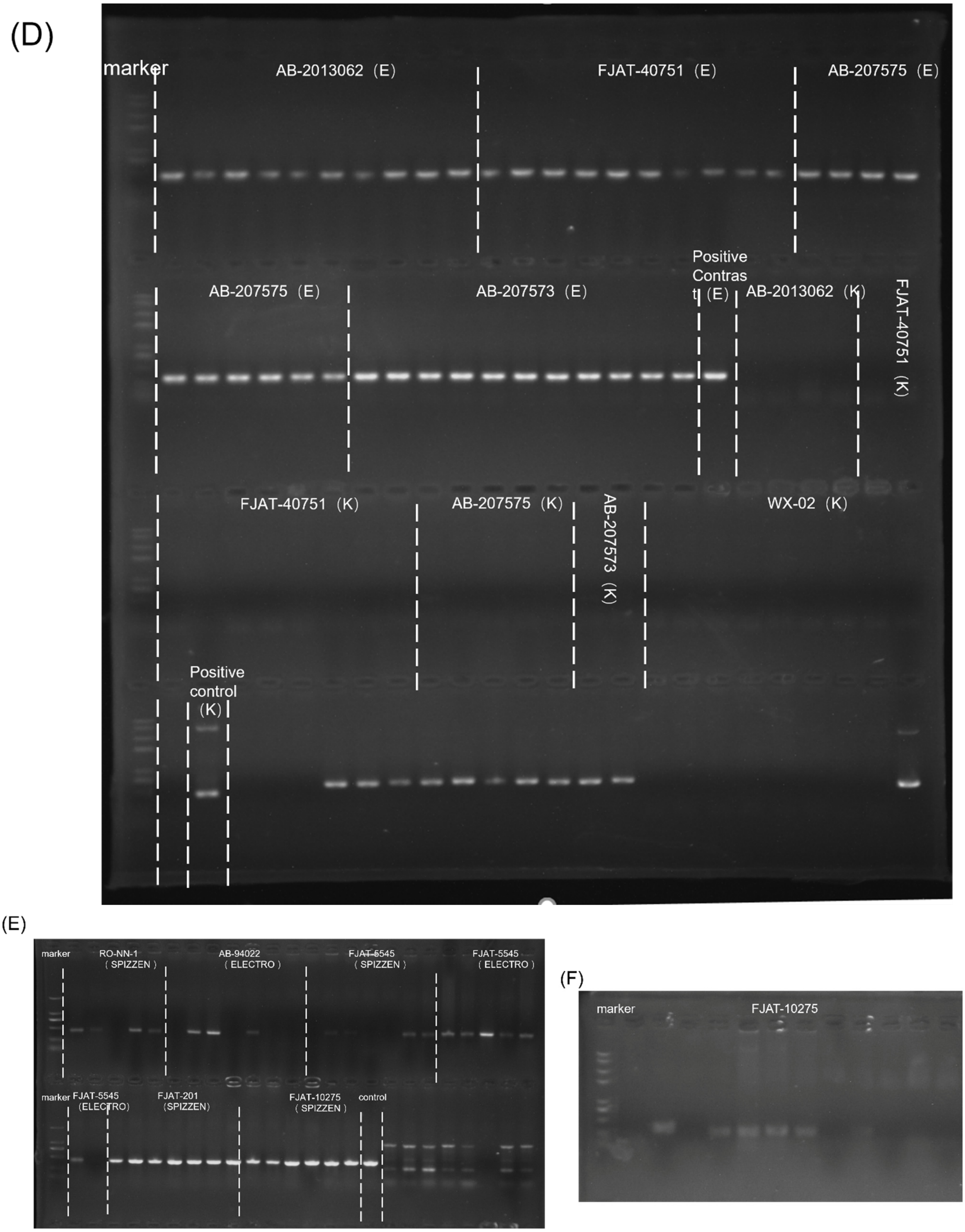

Figure S6. Confirmation of successful plasmid transformation in the wild strains of *B. subtilis* and other bacilli species by CTCNT-P.

(A)PCR test of the plasmid in transformants from CTCNT-P between TLPHMC-G/pBE2 and 15 wild *bacillus* strain.

(B) PCR test of the plasmid in transformants from CTCNT-P between TLPHMC-G/pNNB194 and 15 wild *bacillus* strain.

(C) PCR test of the plasmid in transformants from CTCNT-P between TLPHMC-G/pGK12H and 15 wild bacillus strain.

(D) PCR test of the plasmid in transformants from electro-transformation between TLPHMC-G/pBE2, TLPHMC-G/ pNNB194 and 15 wild bacillus strain.

(E) PCR test of the plasmid in transformants from electro-transformation and Spizzen Transformation between TLPHMC-G/ pNNB194 and 15 wild bacillus strain.

(F) PCR test of the plasmid in transformants from electro-transformation and Spizizen Transformation between TLPHMC-G/ pGK12H and 15 wild bacillus strain., only FJAT-10275 successfully acquired the plasmid.

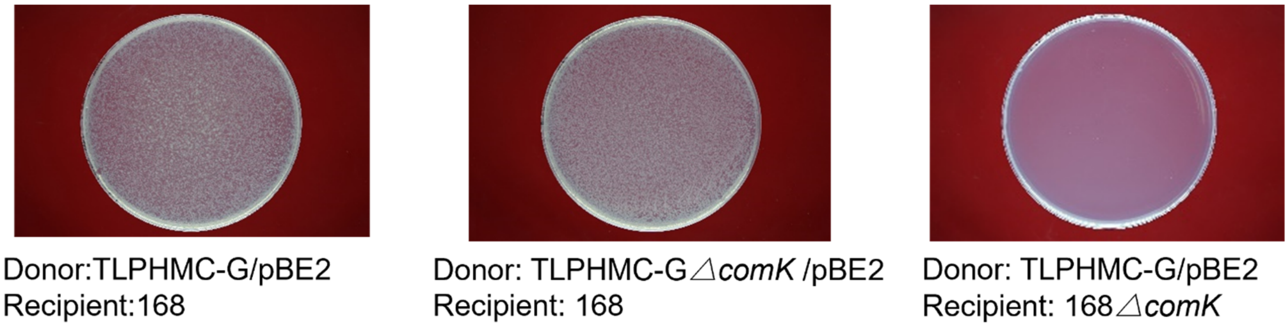

Figure S7. knockouting of the *comK* gene in the donor strain does not affect the CTCNT-P process.

The strain TLPHMC-G Δ*comK*/pBE2 was constructed as the donor strain by knocking out *comK* in TLPHMC-G without any detectable scar and then reintroducing the pBE2 plasmid. Compared with using TLPHMC-G/pBE2 as the donor strain in the CTCNT-P process, no difference in the number of transformants was observed when *Bacillus subtilis* 168 was used as the recipient strain. However, when the recipient strain was replaced with *B. subtilis* 168Δ*comK*, no transformants were produced.This indicates that the *comK* gene is essential for the recipient strain to generate recombinants, but its presence in the donor strain is not necessary for the process.

References

1. Palmer, B.R.; Marinus, M.G. The dam and dcm strains of Escherichia coli—a review. *Gene* **1994**, *143*, 1-12.
2. Kunst, F.; Ogasawara, N.; Moszer, I.; Albertini, A.M.; Alloni, G.; Azevedo, V.; Bertero, M.G.; Bessières, P.; Bolotin, A.; Borchert, S.; et al. The complete genome sequence of the Gram-positive bacterium Bacillus subtilis. *Nature* **1997**, *390*, 249-256, doi:10.1038/36786.
3. Cohan, F.M.; Roberts, M.S.; King, E.C. THE POTENTIAL FOR GENETIC EXCHANGE BY TRANSFORMATION WITHIN A NATURAL POPULATION OF BACILLUS SUBTILIS. *Evolution* **1991**, *45*, 1393-1421, doi:10.1111/j.1558-5646.1991.tb02644.x.
4. Gao, J.; Xie, G.; Peng, F.; Xie, Z. Pseudomonas donghuensis sp. nov., exhibiting high-yields of siderophore. *Antonie Van Leeuwenhoek* **2015**, *107*, 83-94, doi:10.1007/s10482-014-0306-1.
5. Deng, L.; Wang, C.; Zhang, X.; Yang, W.; Tang, H.; Chen, X.; Du, S.; Chen, X. Cell-to-cell natural transformation in Bacillus subtilis facilitates large scale of genomic exchanges and the transfer of long continuous DNA regions. *Nucleic Acids Research* **2023**, *51*, 3820-3835, doi:10.1093/nar/gkad138.
6. Jensen, G.B.; Andrup, L.; Wilcks, A.; Smidt, L.; Poulsen, O.M. The aggregation-mediated conjugation system of Bacillus thuringiensis subsp. israelensis: host range and kinetics of transfer. *Curr Microbiol* **1996**, *33*, 228-236.

7. Wei, X.; Ji, Z.; Chen, S. Isolation of Halotolerant Bacillus licheniformis WX-02 and Regulatory Effects of Sodium Chloride on Yield and Molecular Sizes of Poly-γ-Glutamic Acid. *Applied Biochemistry and Biotechnology* **2010**, *160*, 1332-1340, doi:10.1007/s12010-009-8681-1.
8. Guo, X.; Xiong, Z.; Jia, S.; Xu, Y. The construction of multifunctional shuttle vectors of Bacillus subtilis-Escherichia coli. *Chin J Biotech* **1991**, *7*, 224-229.
9. Kok, J.; van der Vossen, J.M.; Venema, G. Construction of plasmid cloning vectors for lactic streptococci which also replicate in Bacillus subtilis and Escherichia coli. *Appl Environ Microbiol* **1984**, *48*, 726-731, doi:10.1128/aem.48.4.726-731.1984.
10. Nguyen, H.D.; Phan, T.T.; Schumann, W. Expression vectors for the rapid purification of recombinant proteins in Bacillus subtilis. *Curr Microbiol* **2007**, *55*, 89-93, doi:10.1007/s00284-006-0419-5.
11. Bindel Connelly, M.; Young, G.M.; Sloma, A. Extracellular proteolytic activity plays a central role in swarming motility in Bacillus subtilis. *J Bacteriol* **2004**, *186*, 4159-4167.
